# A Novel 18-Norclerodane Diterpenoid from the Roots of *Tinospora sagittata* var. *yunnanensis*

**DOI:** 10.3390/molecules15118360

**Published:** 2010-11-16

**Authors:** Xiang-Zhong Huang, Chun-Mei Cheng, Yun Dai, Guang-Miao Fu, Jun-Ming Guo, Yan Yin, Hui Liang

**Affiliations:** 1 Key Laboratory of Ethnic Medicine Resource Chemistry, School of Chemistry and Biotechnology, Yunnan University of Nationalities, Kunming 650031, China; 2 School of Pharmacy, Henan College of Traditional Chinese Medicine, Zhengzhou 450008, China

**Keywords:** *Tinospora sagittata* var. *yunnanensis*, clerodane diterpenoid, sagitone, cytotoxic activity

## Abstract

A novel 18-nor-clerodane diterpenoid named sagitone (**1**) was isolated from the 95% ethanol extract of dry roots of *Tinospora sagittata* var. *yunnanensis* together with the five known diterpenoids columbin (**2**), palmatoside C (**3**), fibleucin (**4**), tinophylloloside **(5)** and epitinophylloloside (**6**). The structure of the new compound **1** was determined based on MS, IR, 1D and 2D NMR spectral data. The compounds **1~6** did not show significant cytotoxic activity against cancer cell lines K562 and HL-60.

## 1. Introduction

**Figure 1:**
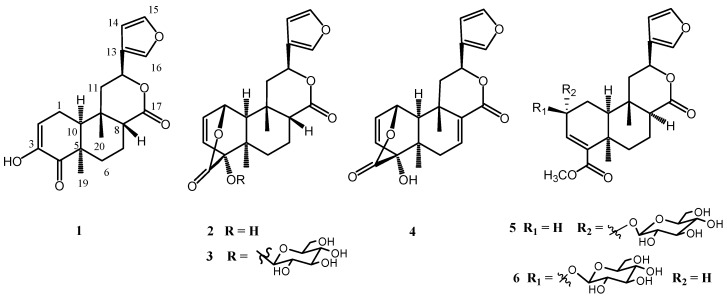
The structures of compounds **1–6**.

The clerodane diterpenoids occupy a unique and important place in the natural product field, because of their widespread distribution, extensive structural variation, and pronounced biological properties such as cytotoxic, antibacterial and antimicrobial activity [1,2,3,4]. Most species of the genus *Tinospora* (Menispermaceae) are among the most widely employed medicinal plants throughout a large part of Asia and Africa, and the characteristic constituents of this genus are the clerodane-based furnoid diterpenoids [5,6]. *Tinospora sagittata* var. *yunnanensis*, a member of family Menispermaceae, is a special plant native to Yunnan and Guangxi Province, China. The roots of *T. sagittata* var. *yunnanensis* has been used for thousands of years in Traditional Chinese Medicine for treating sore throat, laryngitis, gastralgia, and diarrhea [7]. Previous phytochemical investigation on the roots of *T. sagittata* var. *yunnanensis* revealed a number of alkaloids and clerodane-type diterpeneoids [8]. In the present study, we describe the isolation and structure elucidation of a novel 18-nor-clerodane diterpenoid, named sagitone (**1**), together with five known diterpenoids, columbin (**2**), palmatoside C (**3**), fibleucin (**4**), tinophylloloside **(5)** and epitinophylloloside (**6**), from the 95% ethanol extract of the dry roots of *T. sagittata* var. *yunnanensis* (Figure 1). The structure of the new compound **1** was determined based on MS, IR, 1D and 2D NMR spectral data, and the known ones **2**-**6** were identified by comparing their NMR data with those in the literature. All compounds did not show considerable inhibitory cytotoxic activity against cancer cell lines K562 and HL-60 at a concentration of 10 μM.

## 2. Results and Discussion

Compound **1** was obtained as an amorphous powder and with positive reaction in the 10% H_2_SO_4_-EtOH test. The molecular formula was deduced from a pseudo-molecular ion [M + H]^+^ at *m/z* 331.3816 in the HR-ESI-MS (calcd. for C_19_H_23_O_5_, 331.3829), which was in agreement with the ^1^H-NMR, ^13^C-NMR and DEPT spectra (Table 1). The IR spectrum showed characteristic absorption bands for hydroxyl group (3,407 cm^−1^), *δ*-lactone carbonyl (1,710 cm^−1^), *α,β*-unsaturated ketone (1,672 cm^−1^) and furan (874 cm^−1^) functions. Additionally, a strong absorption bond at 238 nm in the UV spectrum also suggested the presence of an *α,β*-unsaturated ketone group.

The ^1^H-, ^13^C-NMR and DEPT spectra of compound **1** (Table 1) revealed 19 carbons, consisting of two methyls, four methylenes, seven methines, and six quaternary carbons. Taking into account the nine degrees of unsaturation, compound **1** should include four rings. Careful analysis of its ^1^H- and ^13^C-NMR data strongly suggested that the compound exhibited typical clerodane diterpenoid signals. The characteristic ^1^H- and ^13^C-NMR signals of **1** revealed the existence of a *β*-substituted furan ring (*δ* 6.42 s, 7.43 s and 7.45 s; *δ* 108.4, 143.8, 139.6, 125.0). The one proton double doublet at *δ* 5.42 (*J* = 12.4, 4.0 Hz) was assigned to the C-12 proton and two one-proton double doublets at *δ* 2.30 (*J* = 14.8, 4.0 Hz) and 1.73 (*J* = 14.8, 12.0 Hz) were attributed to the C-11eq and C-11ax protons, respectively. The presence of a carbinolic carbon was also evident from ^13^C-NMR signal at *δ* 70.6 (C-12). The two methyl groups at C-9 and C-5 were observed as three proton singlets at *δ* 1.30 and 0.96, respectively. The signals at *δ* 2.30 and 2.20 were assigned to the protons at C-8 and C-10, and the C-6 and C-7 methylene protons resonating at *δ* 2.40 (m), 1.19 (dt, *J =* 14.0, 4.0 Hz) and 2.25 (m), 1.61 (m), respectively.

**Table 1:** ^1^H- and ^13^C-NMR data of **1** in CDCl_3_. (^1^H at 400 and ^13^C at 100 MHz; *J* in Hz).

Position	*δ*C	DEPT	*δ*H	HMBC
1	20.8	CH_2_	2.87 (m)	C-3, 5, 9, 10
			2.30 (m)	
2	112.4	CH	5. 86 (m )	C-3, 4, 10
3	145.7	C		
4	198.9	C		
5	44.3	C		
6	29.4	CH_2_	2.40 (m)	C-8, 19
			1.19 (dt, 14, 4.0 Hz)	
7	19.2	CH_2_	2.25 (m)	C-5, 9, 17
			1.61 (m)	
8	49.1	CH	2.30 (m)	C-6, 10
9	36.5	C		
10	44.6	CH	2.20 (m)	C-1, 2, 4, 5, 6, 8, 11
11	40.8	CH_2_	2.30 (dd, 14.8, 4.0 Hz)	C-8, 10, 12, 13
			1.73 (dd, 14.8 12.0 Hz)	
12	70.3	CH	5.42 (dd, 12.4, 4.0 Hz)	C-13, 14, 16
13	125.0	C		
14	108.4	CH	6.42 (s)	C-13, 15, 16
15	143.8	CH	7.43 (s)	C-13, 14, 16
16	139.6	CH	7.47 (s)	C-13, 14, 15
17	171.6	C		
19	28.7	CH_3_	1.30 (s)	C-4, 5, 6, 10
20	26.9	CH_3_	0.96 (s)	C-8, 9, 10, 11

**Figure 2:**
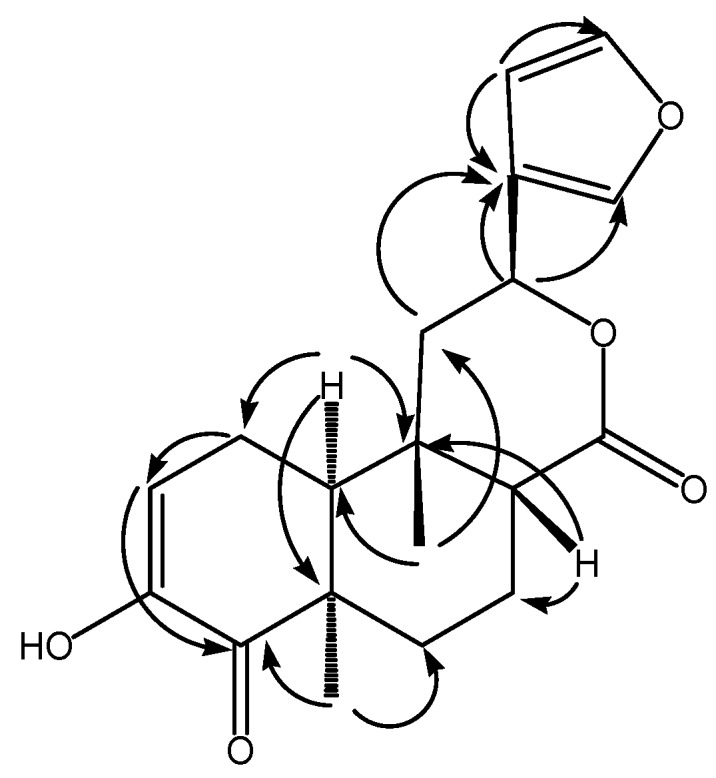
The key HMBC correlations of compound **1**.

The ^1^H‑NMR and ^13^C‑NMR data of **1** are very similar to those of tinocallone C [9] that has been isolated from the roots of *Tinospora capillipes*. The main difference between these two compounds is the chemical shifts of C-2, C-3, and C-4 in ring A in the ^1^H-NMR and ^13^ C-NMR spectra, which are δ 112.4 (C-2), 145.7 (C-3), 198.9 (C-4), and δ 5.86 (m, H-2) in compound **1**, and δ 145.5 (C-2), 128.3 (C-3), 200.8 (C-4), and δ 6.87 (m, H-2), 5.87 (dd, *J* = 10.2, 2.2 Hz, H-3) in tinocallone C [9], indicating that C-3 was substituted by hydroxyl group. In the HMBC spectrum (Figure 2), the correlations between H-2 (δ 5.86, m) and C-4 (δ 198.9), CH_3_-19 (δ 1.30 s) and C-4, H-1 (δ 2.87, 2.30, each m) and C-3 (δ 145.7), H-2 (δ 5.86, m) and C-3(δ 145.7), further confirmed the hydroxyl group at C-3. The position of γ-lactone group was also confirmed by the HMBC correlations observed from H-12 (δ 5.42) to C-13 (δ 125.0), C-14 (δ 108.4) and C-16 (δ 139.6) in the HMBC spectrum. The relative configuration of **1** was deduced by a ROESY analysis. The key correlation between CH_3_-9 and H-8 indicated that these were on the same orientation of the molecule, and were tentatively assumed as *β*-oriented. Similarly, the *α*-orientations of H-10, H-12 and CH_3_-5 were elucidated from the correlations between H-10 and CH_3_-5, and H-10 and H-12. The relative configuration of these positions remained the same as those in tinocallone C [9]. The spectral data of **1** were completely assigned using HSQC, ^1^H-^1^H COSY, HMBC and ROESY experiments. Thus, on the basis of above spectral data, compound **1** was identified as a new 18-norclerodane diterpenoid and named sagitone (Figure 2).

## 3. Experimental

### 3.1. General

All reagents were analytical grade and water was distilled twice. TLC was preformed with silica gel GF_254_ (Marine Chemical Industry Factory, Qingdao, China), and the spots were visualized by spraying with 10% H_2_SO_4_/EtOH reagent. Column chromatography was performed using silica gel (Marine Chemical Industry Factory, Qingdao, China), reverse-phase C_18_ silica gel (Merck, Germany) and Sephadex LH-20 (Sigma). Melting points were measured with an X-4 melting point apparatus and are uncorrected. Optical rotations were recorded on a Perkin-Elmer 241 polarimeter. UV spectra were obtained on a Perkin-Elmer Lambda 900 UV/VIS/NIR spectrophotometer. IR spectra were obtained on a Perkin-Elmer 577 spectrometer with KBr pellets. NMR Spectra were recorded on a Bruker AV-400 spectrometer (^1^H, 400 MHz; ^13^C, 100 MHz), and chemical shifts are presented as values relative to tetramethylsilane as an internal standard. Low-resolution electrospray-ionization mass spectrometry (ESI-MS) and HR-ESI-MS were recorded on a Finnigan LCQ-Advantage mass spectrometer and a VG Auto-Spec-3000 mass spectrometer.

### 3.2. Plant material

The dried roots of *Tinospora sagittata* var. *yunnanensis* were collected in November 2007 from Honghe County, Yunnan Province, China. The plant was authenticated by professor Shao-Bin Ma, Department of Biology, Yunnan University. A voucher specimen (TSY200711) was deposited in the herbarium of the School of Chemistry and Biotechnology, Yunnan University of Nationalities, Kunming, China.

### 3.3. Extraction and isolation

The dried roots (10.0 Kg) of *T. sagittata* var. *yunnanensis* were extracted with 95% ethanol (60 L × 3) for 48 h each at room temperature, and combined extract was concentrated under vacuum to give a brown residue (1,000 g). The residue was then suspended in water (1.5 L) and extracted with EtOAc (1.5 L × 5), and *n*-BuOH (1.5 L × 5) to give the corresponding EtOAc (250 g), *n*-BuOH extract (380 g), and water extract (350 g), respectively.

A part of EtOAc extract (75 g) was subjected to silica gel column chromatography and eluted with a gradient of petroleum ether-MeOH (50:1–0:1, v/v) to afford five fractions (Fr. 1–5). Fr. 3 (5.0 g) was chromatographed on Sephadex LH-20 column using CHCl_3_-MeOH (1:1, v/v) as eluent to give compound **1** (15 mg).

A part of the *n*-BuOH extract (50 g) was subjected to silica gel column chromatography using a stepwise gradient elution of CHCl_3_-MeOH (20:1–2:1) to obtain eight fractions (Fr. 1–8). Fr. 1 (8.0 g) was further purified over Sephadex LH-20 column eluted by MeOH, and then recrystallized in CHCl_3_ to give compound **2** (13 mg). Fr. 2 (6.2 g) was rechromatographed over silica gel column eluted by gradient EtOAc-MeOH (1:0–10:1) to afford compounds **5** (120 mg) and **6** (80 mg). Fr. 3 (4.7 g) was subsequently subjected to Sephadex LH-20 (eluted with MeOH) and ODS (eluted with 1% MeOH in water ) column chromatography, followed by purification over silica gel column chromatography with EtOAc-MeOH (1:0–6:1) as mobile phase to yield compound **3** (50 mg). Compound **4** was collected from Fr. 5 (5.8 g) by ODS (eluted with 20% MeOH) and silica gel column chromatography (eluted with EtOAc-MeOH; 1:0–8:1).

### 3.4. Characterization of compound ***1***

*Sagitone* (**1**): Amorphous powder, M.p. 185–186 °C; [α]^25^_D_= −4.8° (*c* = 0.68, CHCl_3_); UV (MeOH): λ_max_ (log ε_max_): 238 (1.86), 325 (1.26) nm; IR (KBr): ν = 3,407, 3,148, 1,710, 1,672, 1,655, 1,503, 1,403, 1,300, 1,164, 1,082, 874 cm^−1^; ESI-MS: *m/z* = 331 [M + H]^+^, 683 [2M + Na]^+^; HR-ESI-MS: *m/z* = 331.3816 (calcd. 331.3829 for C_19_H_23_O_5_, [M + H]^+^), 683.2856 [2M + Na]^+^; ^1^H- and ^13^C-NMR: see Table 1.

### 3.5. Bioassay

Inhibition of cell-growth activity was determined by a MTT assay using human chronic myelogenous leukemia cells (K562) and human promyelocytic leukemia cells (HL-60) as previously described [10]. c*is*-Diamminedichloroplatinum (DDP) was used as a positive control. None of the compounds showed any obvious cytotoxic effect against cancer cell lines K562 and HL-60 at a concentration of 10 μM.

## 4. Conclusions

Repeated column chromatography (including normal-phase and reverse-phase silica gel, and Sephadex LH-20) of the dry roots of *T. sagittata* var. *yunnanensis* has led to the isolation of a new clerodane-type diterpenoid, named sagitone (**1**), together with the five known diterpenoids columbin (**2**), palmatoside C (**3**), fibleucin (**4**), tinophylloloside **(5)**, and epitinophylloloside (**6**). The structure of the new compound **1** was determined by spectroscopic methods including 1D NMR, 2D NMR and MS experiment, and the structures of the known compounds including **2**~**6** were identified by comparing their NMR data with those of ones in the literature. None of the compounds showed any significant cytotoxic activity against the cancer cell lines K562 and HL-60 at a concentration of 10 μM.
